# Effect of biochar addition on leaf-litter decomposition at soil surface during three years in a warm-temperate secondary deciduous forest, Japan

**DOI:** 10.1038/s41598-019-53615-2

**Published:** 2019-11-18

**Authors:** Yukiya Minamino, Nobuhide Fujitake, Takeshi Suzuki, Shinpei Yoshitake, Hiroshi Koizumi, Mitsutoshi Tomotsune

**Affiliations:** 1 Graduate School of Agricultural Science, Kobe University, 1 Rokkodai, Nada, Kobe, Hyogo, 657-8501 Japan; 2 Takayama Field Station, River Basin Research Center, Gifu University, 919-47, Iwaimachi, Takayama, Gifu, 506-0815 Japan; 3 Faculty of Education and Integrated Arts and Sciences, Waseda University, 2-2 Wakamatsucho, Shinjuku-ku, Tokyo, 162-8480 Japan

**Keywords:** Ecosystem ecology, Forest ecology

## Abstract

The addition of biochar to the forest floor should facilitate efficient carbon sequestration. However, little is known about how biochar addition effects litter decomposition, which is related to carbon and nutrient dynamics in forest ecosystems. This study evaluated the effect of biochar addition on leaf litter decomposition in a forest ecosystem. To examine whether leaf litter decomposition was stimulated above and below biochar, litterbag experiments were carried out for about 3 years in a field site where biochar was added at the rate of 0, 5 and 10 t ha^−^¹ (C0, C5 and C10 plots) to the forest floor in a temperate oak forest, Japan. Biochar addition at C10 significantly enhanced litter decomposition below biochar for 2 years after treatment and above biochar for 1 year after treatment. Litter water content in biochar plots tended to increase under dry conditions. Biochar addition enhanced litter decomposition because of increased microbial activity with increased moisture content and accelerated the decomposition progress rather than changing the decomposition pattern. However, the carbon emission through changing leaf litter decomposition was small when compared with the carbon addition by biochar, indicating that biochar could be an effective material for carbon sequestration in forest ecosystems.

## Introduction

Biochar is a product of thermal degradation of organic matter like plant residues in the absence of oxygen^1^. Biochar was used as a soil conditioner for increasing plant productivity and harvest of crops in agricultural ecosystems in ancient times^2^. Biochar is carbon-enriched, in most cases more than 60%, and is recalcitrant to decomposition in comparison to the original biomass, and hence can remain in the pedosphere for 100–1000 years^3–5^. Biochar addition to ecosystems therefore has great potential in terms of carbon sequestration^1^. Forest ecosystems would be expected to be effective targets for carbon sequestration, because they have high productivity and occupy vast areas of terrestrial ecosystems.

The increase in plant productivity is caused by physical and chemical changes in the pedosphere (soil porosity and water holding capacity, soil pH, cation exchange capacity, and nutrient availability) through biochar addition^6–8^. In addition, it was also reported that biochar addition changes the microbial community composition and stimulates microbial activity through these physical and chemical changes^9^. These changes in the microbial community were associated with enhanced decomposition of soil organic matter (SOM) in some forest ecosystems^10–12^. The potential for carbon sequestration within an ecosystem would be decided by the balance of improving the carbon fixation ability of plants, increasing the soil carbon stock as biochar, and enhancing the decomposition of organic matter. Nevertheless, there is little research about the effect of biochar addition on SOM decomposition in forest ecosystems.

The pedosphere of forest ecosystems is composed of distinct layers, such as organic and mineral soil layers. In particular, the organic soil layer, which is located on the soil surface and is constructed mainly of leaf litter, is enriched with labile carbon and is likely decomposed faster than the bulk SOM. Litter decomposition is controlled by litter properties (chemical components), microbial and fauna properties (community composition and activity), and environmental conditions (temperature and moisture)^13^. Some studies have reported that biochar addition would improve soil environmental conditions such as pH, aeration and water holding capacity, and change the microbial activity, community composition and diversity^3,9^. Therefore, litter decomposition is predicted to be most affected by biochar addition to the forest floor.

Abiven and Andreoli^14^ reported that under controlled conditions charcoal did not promote litter decomposition. In addition, the application of biochar decreased net carbon dioxide (CO_2_) mineralization from mixtures of soil and willow litter by 20% over a 90-day period in a laboratory experiment^15^. Because these studies were performed under controlled conditions, these impacts of biochar might be different from the impacts under field conditions. In contrast, in field experiments, CO_2_ fluxes from the soil surface, including the litter layer, increased with biochar addition in boreal forest^16^. This result indicated that litter decomposition might be enhanced by biochar addition although it was not distinguished whether the released CO_2_ was derived from the litter layer or the mineral soil layer. In addition, fire-derived charcoal might have enhanced the decomposition of fine larch roots in a litterbag experiment in boreal forest^17^. This result would suggest that charcoal might enhance the decomposition of labile organic matter associated with plant residues in the field. It is unclear, however, how biochar addition effects litter decomposition during long-term field experiments in forest ecosystems.

We hypothesized that biochar addition would enhance litter decomposition through changes in soil physiological and biological conditions in the litter layer. To test this hypothesis, different amounts of biochar were added into the forest ecosystem floor of temperate oak forest in Japan. We investigated whether biochar addition impacted on leaf litter decomposition for about 3 years.

## Materials and Methods

### Study site

The experiment was carried out in a deciduous secondary forest of Honjo Waseda Research Park, Saitama, Japan (36°12'57” N, 139°10'18” E). The area is categorized as warm temperate, with annual mean temperature of 15.0 °C and a total annual precipitation of 1286.3 mm (1981–2010). The forest is dominated by oak (*Quercus serrata*), the tree density on the site was 383 trees ha^−^¹, and the understory vegetation is bamboo grass (*Pleioblastus chino*). The soil was originally derived from alluvion volcanic ash, classified as Alic Hapludands.

### Experimental plots

Biochar was spread manually on the top of the organic layer at rates of 0, 5, and 10 t ha^−1^ (C0, C5, and C10 plots, respectively). Each treatment was applied to three replicate plots of 20 m × 20 m in November 2015. The biochar (particle size <5 mm) was made of oak wood chips at 600–700 °C (Shiratori super MOKUTAN C, Shiratori Mokuzai Kakoh Cooperative Society, Japan). The proportion of carbon in biochar was about 71%, and then 3.6 and 7.1 tC ha^−1^ was used as the amount of carbon input to C5 and C10 plots, respectively. Aboveground biomass was not significantly different among C0, C5 and C10 plots at the time of biochar addition (p > 0.05).

### Litterbag experiment

The litterbag method was used to clarify the litter decomposition rate. Litterbags (15 × 15 cm) were made of nylon cloth with a mesh size of approximately 2 mm. Two kinds of leaf litter (3 g dry weight of each) of *Q. serrata* were placed in litterbags. One was made with leaf litter collected in November 2015, which was new leaf litter that had fallen after biochar addition (new litter: NL), the other was made with leaf litter collected in August 2015, which was leaf litter located under the biochar layer after biochar addition (old litter: OL). After sampling, the litters were air-dried until the beginning of the experiment. In January 2016, NL and OL litterbags were placed above and below the biochar layer, respectively. Sampling of the bags took place 9 times: 85, 180, 270, 360, 470, 553, 673, 840, and 932 days after initiation of the experiment. At each sampling timepoint, three each of the NL and OL litterbags (n = 3) were collected from 9 subplots.

### Chemical analysis

Fresh litter mass was measured after samples were taken in litterbags. Thereafter, litter samples were dried at 105 °C for 24 h, and dry litter mass was measured after removing contaminating root and soil material. Litter water content (%) was calculated by the difference in weight between fresh and dry litter. A portion of litter sample was heated at 550 °C for 6 h, and the residue weight was measured as ash. Ash was measured to correct contamination of soil and biochar, and ash-free remaining mass (%) Ash‐free remaining mass (%) was calculated following Eq. (1) below:

1 $$\mathrm{Ash}-\mathrm{free}\,{\rm{remaining}}\,{\rm{mass}}\,( \% )=\frac{{{\rm{M}}}_{{\rm{t}}}(1-{{\rm{A}}}_{{\rm{t}}})}{{{\rm{M}}}_{0}(1-{{\rm{A}}}_{0})}\times 100$$

where M_t_ is the litter mass (g) after a given period, M_0_ is the initial litter mass (g), A_t_ is the litter ash content (%) after a given period and A_0_ is the initial litter ash content (%).

Acid-unhydrolyzable residue (AUR) and total carbohydrate were measured by the Klason method as in Osono *et al*.^18^. In brief, samples were washed with alcohol-benzene (ethanol:benzene = 1:2 (v/v)) and the residue was hydrolysed with 72% and 2.5% sulfuric acid (v/v) in sequence. After hydrolysis, the residue was filtered and weighed as AUR. The filtrate was used for total carbohydrate analysis by phenol-sulfuric acid methods. Lignin was defined as the weight of ash of AUR deducted from the total weight of AUR.

Estimation of the carbon balance between biochar addition and changing litter decomposition

To compare the carbon emission through changing leaf litter decomposition (induced carbon emission) with the carbon addition by biochar (biochar carbon) on an area basis, the decomposed litter mass (tC ha^−1^) in the experimental period was estimated from three components: initial litter mass under the field condition, litter decomposition ratio and carbon concentration. The initial litter mass (td.w. ha^−1^) of NL was the average annual dry weight of leaf litter collected from 5 litter traps in each plot in 2015. The values for OL were obtained from the values of the initial litter mass of NL multiplied by the ratio of litter remaining of NL at 360 days (about one year) in the C0 plot using the litter bag method. The litter decomposition ratio used the values at 932 days (last sampling day, about three years) measured by the litter bag method. Carbon concentration was measured by CHNS/O analyser (PE2400, PerkinElmer, USA) for initial samples. Induced carbon emission was the difference in the decomposed litter mass between control and biochar treatment plots.

### Data analysis

All data were expressed as means ± standard errors. A one-way analysis of variance (ANOVA) followed by Tukey’s Honestly Significant Difference test was used to compare the remaining litter mass of the different rates of biochar addition (p < 0.05). T-test was used to compare the difference in litter water content between litter with and without biochar. Statistical analysis of the data was carried out with SPSS 17.0 Statistics (IBM Corp., Armonk, NY, USA). Ash-free accumulated mass loss vs substrate remaining mass was fitted based on a single exponential model y = ae^(−x/b)^ + c^19,20^.

## Results

**Figure 1:**
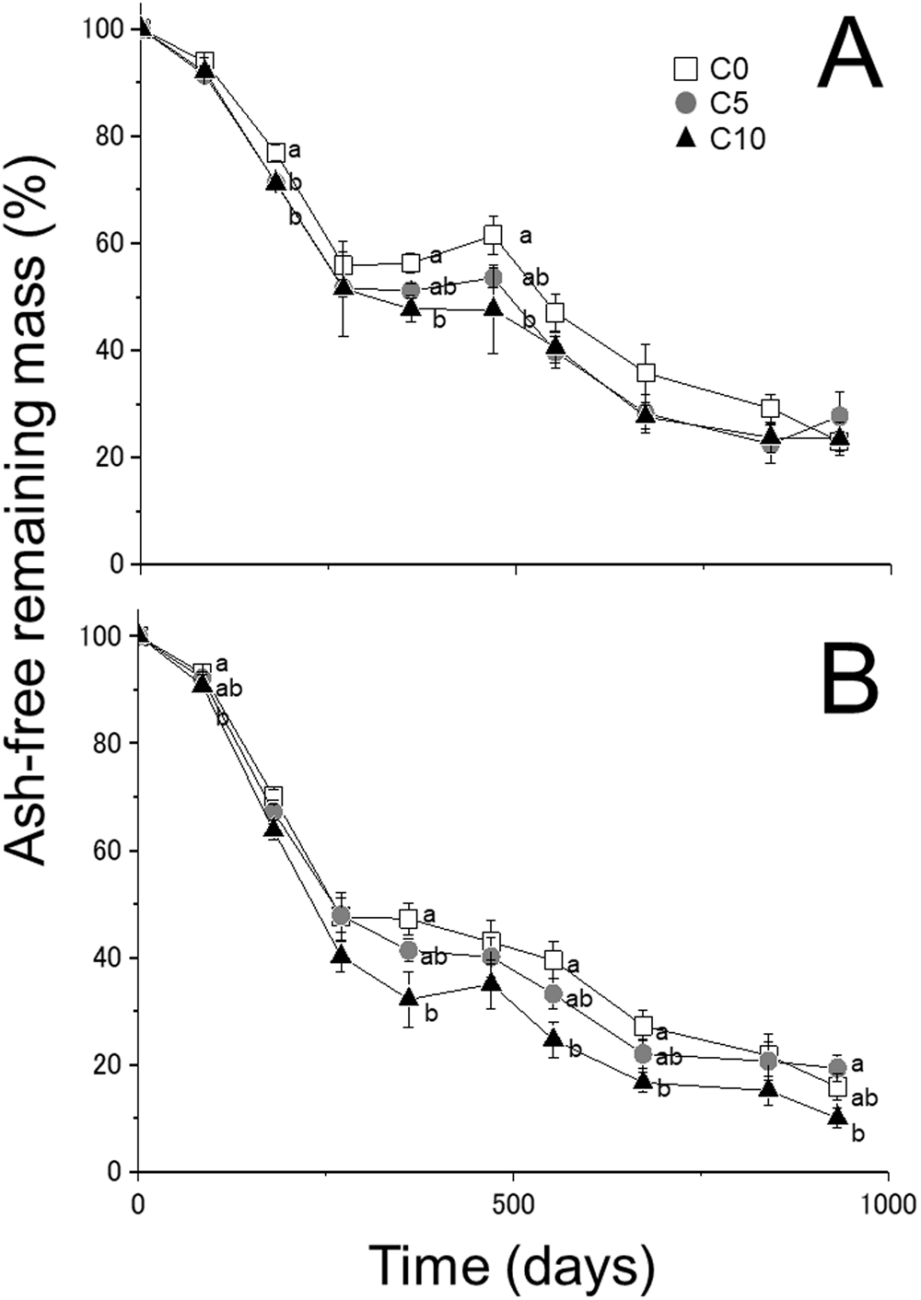
Change in remaining mass in (**A**) new litter (NL) and (**B**) old litter (OL) over 932 days at 0 t ha^−1^ (C0), 5 t ha^−1^ (C5) and 10 t ha^−1^ (C10) biochar. Symbols show means and bars indicate standard errors (n = 9). Different letters indicate statistically significant differences (p < 0.05) between biochar addition rates.

For NL, the ash-free remaining mass tended to decrease with increasing rates of biochar addition until 470 days, with significant differences between C0 and C10 at 180, 360, and 470 days (Fig. 1A). In the period from 553 to 932 days, however, ash-free remaining mass was not significantly different among plots with and without biochar (Fig. 1A). For OL, the ash-free remaining mass tended to decrease with increasing rates of biochar addition until 673 days, with significant differences between C0 and C10 at 85, 360, 553, and 673 days (Fig. 1B).

**Figure 2:**
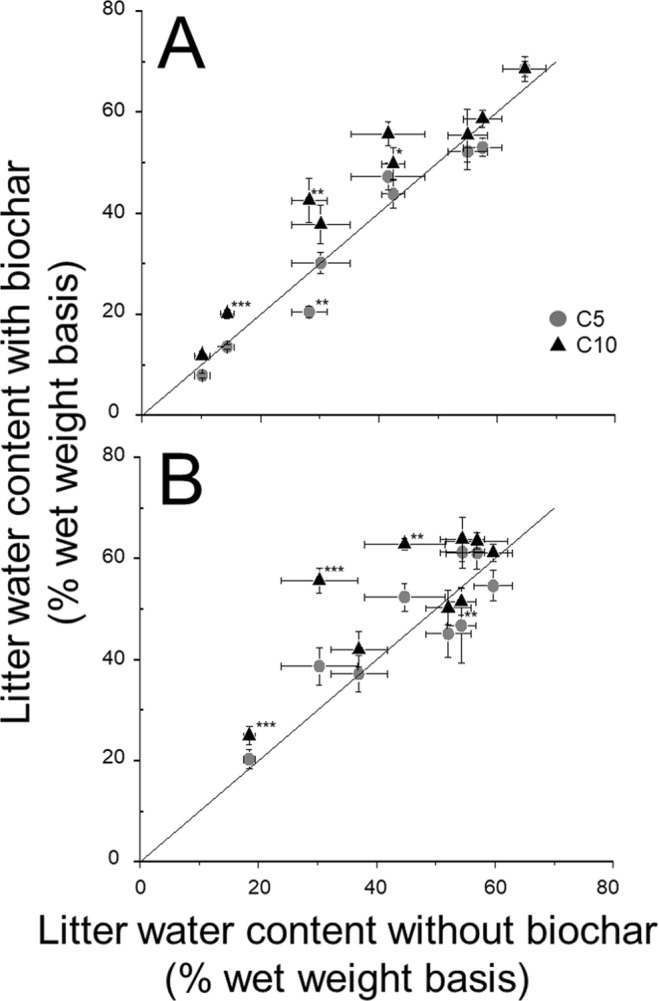
Litter water content in (**A**) new litter (NL) and (**B**) old litter (OL) either in the absence: 0 t ha^−1^ (C0) or presence: 5 t ha^−1^ (C5) and 10 t ha^−1^ (C10) of biochar. Symbols show means and bars indicate standard errors (n = 9). The solid line represents the theoretical 1:1 line where the litter water content with and without biochar is equal. Asterisks indicate significance of differences from the solid line: *p < 0.1, **p < 0.05, ***p < 0.01.

When litter water content in C0 was >50%, litter water content was not significantly different irrespective of biochar addition (Fig. 2). In contrast, when litter water content in C0 was <50%, litter water content in biochar addition plots tended to be higher than that in C0 (Fig. 2). The litter water content in C10 was clearly different from that in C0, and in some cases significantly different (p < 0.05).

**Figure 3:**
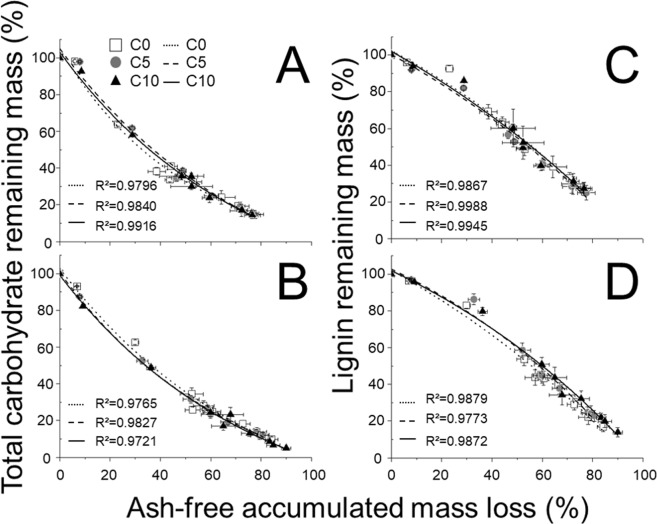
Change in total carbohydrate (left) and lignin (right) remaining mass with accumulated mass loss of litter in (**A**), (**C**) new litter (NL) and (**B**), (**D**) old litter (OL) at 0 t ha^−1^ (C0), 5 t ha^−1^ (C5) and 10 t ha^−1^ (C10) biochar. Symbols show means and bars indicate standard errors (n = 9). All fitting curves were significant (p < 0.01).

For total carbohydrate, the fitting curves tended not to be different among C0, C5 and C10 with respect to NL and OL (Fig. 3A,B). For lignin, the fitting curves tended not to be different among C0, C5 and C10 with respect to NL and OL (Fig. 3C,D). Although ash-free accumulated mass loss in OL tended to be larger than that in NL, the fitting curves tended not to be different between NL and OL. For NL and OL during litter decomposition, total carbohydrate was decomposed first, thereafter lignin was decomposed (Fig. 3).

The decomposed litter mass estimated from initial litter mass, decomposition rate and carbon concentration, tended to be higher in C5 and C10 than in C0 during the experimental period (Table 1). The amounts of induced carbon emission were −0.1 and 0.3 tC ha^−1^ in C5 and C10 plots, respectively. The amount of biochar carbon was 3.6 and 7.1 tC ha^−1^ in C5 and C10 plots, respectively, and therefore the ratios of induced carbon emission to biochar carbon were −1.5 and 4.0% in C5 and C10 plots, respectively.

**Table 1:** Estimation of the balance between carbon emission through changing leaf litter decomposition and carbon addition by biochar.

		Treatment		
		C0	C5	C10
Initial litter mass (td.w. ha^−1^)	NL	6.4	6.6	6.7
	OL	3.8	3.8	3.8
Litter decomposition ratio (%)	NL	70.7	70.0	72.2
	OL	80.3	74.7	87.3
Litter carbon concentration (%)	NL	46.4	46.4	46.4
	OL	45.5	45.5	45.5
Decomposed litter mass (tC ha^−1^)	NL	2.1	2.1	2.3
	OL	1.4	1.3	1.5
	Total	3.5	3.4	3.8
Induced carbon emission (tC ha^−1^)		—	−0.1	0.3
Biochar carbon (tC ha^−1^)		—	3.6	7.1
Ratio (%)		—	−1.5	4.0

C0, C5 and C10 indicate the experimental plots with 0, 5 and 10 t ha^−1^ of biochar carbon, respectively. OL and NL indicates old and new litter that has fallen before and after biochar addition, respectively.

## Discussion

Our results suggested that biochar addition has the potential to stimulate decomposition of leaf litter that is in direct contact with the biochar (Fig. 1). The stimulation could be caused by both abiotic and biotic factors. Prescott^21^ found that moisture and temperature conditions could control litter decomposition by meta-analysis of global research. Recent studies have shown that biochar has high water holding capacity, and that soil with biochar addition also had increased water holding capacity and water content in forest ecosystems^7^. For example, Prober *et al*.^22^ reported that soil moisture content in temperate woodlands increased by 6–25% after biochar addition. Our study also found that litter water content increased with biochar addition, especially under dry conditions (Fig. 2). This result would be caused by the humidity control function of biochar^23,24^. In general, the litter layer is easily dried because it is located at the soil surface. Litter in biochar plots, however, would maintain high water content constantly because the high water holding capacity of biochar could maintain fixed humidity within the litter layer^25^. The result that biochar enhanced litter decomposition in this study corresponded with the results in other field experiments^7,17^, however, it did not correspond with the results in laboratory experiments^14,15^. This discrepancy might be related to the avoidance of dry conditions by the controlled conditions in laboratory experiments.

Some previous studies reported that biochar could also affect the microbial community, leading to enhanced decomposition of soil organic matter^9,26^. In addition, some studies reported that differences in microbial community composition were associated with changed litter decomposition rate and decomposition pattern in temperate ecosystems^27,28^. The drivers of litter decomposition could be acceleration of decomposition progress by increasing microbial activity, or changes in the decomposition pattern by changing microbial community composition. In this study, the decomposition pattern, which was characterized by the fitting curve of ash-free accumulated mass loss vs substrate remaining mass, was not different among treatments (Fig. 3). This result indicated that litter decomposition might be enhanced by accelerating decomposition progress rather than changing decomposition pattern. Our results that first carbohydrate and then lignin was decomposed, corresponded with those of Berg^29^.

Lehmann and Sohi^30^ reported that the priming effect of labile carbon in biochar might enhance the decomposition of SOM. Biochar produced at low temperature (250–400 °C) contained a substantial labile fraction and increased the soil respiration rate^31,32^. In this study the biochar was produced at relatively high temperature (600–700 °C), which suggests that there would be little labile carbon and therefore the priming effect would not significantly impact litter decomposition^33^. In addition, biochar usually has an alkaline pH, leading to an increase in pH when applied to the mineral soil layer; its application can also enhance the availability of nutrients such as nitrogen and phosphorus because of its highly porous structure^16,34^. Some previous studies^35–37^ reported that these effects described above could accelerate the decomposition progress and change the decomposition pattern of organic matter. However, pH measurement of the litter layer is not common, therefore it was not clear whether these factors were responsible for enhanced litter decomposition in our study.

Our results indicated that OL showed enhanced decomposition with biochar addition for 2 years, but NL showed enhanced decomposition only for 1 year (Fig. 1). The difference in litter water content between C0 and the biochar plots in OL was higher than that in NL, and high litter water content was maintained in OL. From these results, litter decomposition below biochar would be more affected than that above biochar. This might be related to the supply of nutrients leached from biochar^38^, or because the contact between biochar and the litter layer below is more intimate than that of the litter layer above because of gravity. These results might suggest that organic matter decomposition below the biochar layer is important for carbon sequestration. However, the decomposition process was not significantly different between NL and OL (Fig. 3).

In our experimental period, the carbon emission through changing leaf litter decomposition was very small when compared with the carbon addition by biochar. This result indicates that biochar could be an effective material for carbon sequestration in forest ecosystems. In general, mass loss from litter bags include mineralization, leaching and fragmentation processes, although it is difficult to apportion the losses to these three processes and to quantify only the carbon emission to the atmosphere^39^. Previous studies^29,40^ reported that soluble components accounting for up to 30–40% of leaf mass could be lost through the leaching process. In addition, Lecerf^39^ reported that 26% of oak leaf litter mass was lost by fragmentation using the various mesh size of litter bags. According to these previous results, about half of the induced carbon emission based on mass loss was roughly regarded as carbon emission to the atmosphere, and the ratios of atmospheric emission to biochar carbon were very small (−0.8% in C5, 2.0% in C10).

In conclusion, biochar addition stimulated litter decomposition in deciduous temperate forest for about 3 years. It was caused by improvement of water conditions and microbial activity. However, the carbon emission through changing leaf litter decomposition was small when compared with the carbon addition by biochar. This result indicates that biochar could be an effective material for carbon sequestration in forest ecosystems.
